# Embodied agency through soft skills development in dance

**DOI:** 10.3389/fcogn.2024.1396904

**Published:** 2024-08-01

**Authors:** Sara Houston

**Affiliations:** School of Arts, Humanities and Social Sciences, University of Roehampton London, London, United Kingdom

**Keywords:** dance, Parkinson's disease, embodied agency, soft skills, social justice

## Abstract

The fluidity, adaptability and complexity of a dancer's movement are often used as examples of how dance at a level of mastery is embodied. The freedom this gives the dancer to choose what and how they move is enjoyed at a subconscious level, with often tacit knowledge driving the artistic and technical brilliance. The topic of embodied agency in dance becomes more complicated when examining dance for the older person who has had little training. Embodiment is not straight forward, particularly if one examines dancing for the older adult with a neurodegenerative condition, which disrupts cognition and physical movement. Yet, as I argue, embodied agency is a key vision for socially engaged dance practices, particularly using improvisation. In the paper I examine how centring the amateur older dancer with a neurological condition means looking again at embodied agency and what it could mean in this context, as well as what the conditions are that might make it work. The paper takes the example of Dance Well, a group of community dancers in Italy that accommodates people with Parkinson's and others, including those seeking asylum. With this example I draw upon ideas from research I co-led to name some of the tacit soft skills—such as empathy and understanding and appreciating difference—developed through Dance Well's engagement with the local community, which, I now suggest, led to a process of embodied agency. I argue that in identifying this anoetic knowing, it is clear that embodied agency is not just about *mastery* of movement, but about important relational skills that are embodied and practiced through dancing, even by those with little formal dance training. I argue that moving together whilst embodying soft skill qualities may nurture an environment that could enable the transformation of relationships between those dancing and contribute to the creation of an important and meaningful activity within the community. In this way, the paper outlines ideas on how embodied agency through dancing may contribute to a vision of social justice and a characterization of embodiment that emphasizes the recognition of each other's humanity.

## Introduction

In this article I focus on the idea that embodied agency can be harnessed through the practicing of soft skills in the context of a specific dance programme that invites people with Parkinson's and others to share creative movement. The article is divided into sections that take the reader through my argument. First, I lay out my definitions of both soft skills and embodied agency and contextualize them in relation to the current literature and disciplinary foci, as well as in relation to dance. I suggest that there is a gap in knowledge in linking both embodied agency and soft skills together within the discipline of Dance Studies, and when examining dance practices. Second, I outline the project, Empowering Dance and its case study of Dance Well: Movement Research for Parkinson, which gave impetus and evidentiary grounding to my thoughts in this area. I then go on to examine the concept of embodied agency as it currently pertains to dancing in a professional context, and I outline my counterargument following this, seeing the problems for community dancers, particularly those with Parkinson's, and I suggest a different approach through the lens of soft skills. I use the case study of Dance Well to expand on this argument and conclude that understanding embodied agency through soft skills could lead to appreciating the contribution dancing might make to a vision of social justice and a characterization of embodiment that emphasizes the recognition of each other's humanity.

## Introduction to soft skills and embodied agency

Soft skills are the patterns of thought, behaviors and qualities that enable interpersonal relationships to thrive and support the navigation of personal emotions. The article is particularly interested in the connection between the idea that soft skills can emerge and develop within some dance practices, particularly in community settings, and the impact of dancing on all involved, which crucially might include a development of embodied agency. The notable quality of many soft skills is that, because the body plays a central role in navigating emotions and the dynamics of interpersonal exchange, they often emerge in and grow through the body and by encountering others in space. There is therefore a potentially interesting relationship between dancing—the playful, creative and aesthetic movement of the body as it connects to others moving—and soft skills, the embodied support for relationships to flourish. The article goes further in suggesting that whilst it would not necessarily be common to talk about embodied agency in relation to community dancers, who dance for fun rather than professionally, embodied agency could be the element of impact for community dancers that is felt through a dance process where soft skills are championed.

As a starting point for thinking about embodied agency, I take Noland's (2009) definition. She writes that embodiment is,

the process whereby collective behaviors and beliefs, acquired through acculturation, are rendered individual and “lived” at the level of the body. *Agency* it follows, is the power to alter those acquired behaviors and beliefs for purposes that may be reactive (resistant) or collaborative (innovative) in kind (Noland, 2009, p. 8–9).

I take the idea that collective behaviors and beliefs are seen within the sub-cultures formed through dance practices, as well as in everyday activities. It should be noted again here that soft skills also concern behaviors and thoughts cultivated within practices and sub-cultures.

Embodiment is a large expansive topic tackled from various fields of thought. In particular, there has been strong representation from work within the Sociology of the Body, with Shilling (for example, Shilling, 1997, 2013, 2016; Wacquant, 2004; Aalten, 2007; Crossley, 2007; Turner, 2008; Frank, 2013). Some of this work has attended explicitly to dance case studies, seeing it as a physical and embodied activity within a wider social system. The main thrust of this work has been to counteract traditional schools of thought in Sociology that have neglected the body's role within social systems and activities. It is no co-incidence that Dance Sociologists, such as Thomas (1993, 2003), reiterate this claim as it pertains to the marginalization of dance within society and within the arts. Key within Sociological and Anthropological thought has been the seminal work of Mauss (1935) whose work on body techniques set the stage for expanded thinking within the area of embodiment (for example through Crossley's work).

The areas of Cognitive Science and Neuroscience have also contributed to the concept in examining the neural mechanisms for conditions of embodiment and the work of Calvo-Merino, Jola, Glaser and Haggard (for example Calvo-Merino et al., 2008 and separately) is key to this area in looking at professional dancers, as is Batson and with Wilson's (2014) work examining cognition's role in Somatics. Within Dance Studies Grau's (2005, 2012) work in the Anthropology of Dance stands out for its sensitivity to differing cultural conceptions of body, embodiment and dance and how embodiment of the moving body is crucial to understanding certain peoples and ways of life. Philosophy of Dance has been key in its examination of the concept of embodiment and the experience of dancing, with Sheets-Johnstone (1966), Fraleigh (1987), Parviainen (2002), Warburton (2011), Katan (2016), and Bresnahan et al. (2020) as examples of authors who have introduced dance into philosophical disciplines, such as phenomenology and epistemology. From a Cultural Studies angle, Noland (2009) has in particular been useful in expanding the discussion on embodied agency, gesture and dance explicitly.

Soft skills as a subject area has had attention from different disciplines to the ones covering embodiment and embodied agency. Most of the thought on this topic has emanated from Business Studies and Management Studies (see for example Andrews and Higson, 2008; Kautz et al., 2014; Yoke and Ngang, 2015; Marin-Zapata et al., 2022) where the debate has been to define the term and to examine how soft skills may help in leadership training and leadership success, as well as how to train business students and early career graduates in soft skills. Additionally, the European Union has commissioned research on the topic of soft skills, with a view to incorporating soft skills into policy (see for example, Council of Europe, 2016; Rodrigues et al., 2021), as have other international bodies, such as OECD (2018) and UNICEF (Hoskins and Liu, 2019). The interest in soft skills to be integrated in high level workforce development policy was underlined by the 2023 report by the World Economic Forum, which declared that soft skills—such as negotiation, understanding and appreciating difference, care and patience—were the most important set of skills in the workplace now and in the future. The Organization for Economic Co-operation and Development (OECD) went further arguing that to navigate a complex and uncertain world, soft skills are vital (OECD, 2018).

Despite this interest at policy and academic levels, soft skills (sometimes called life skills, or 21st century skills) have hardly been recognized in dance practice and scholarship, despite these skills being used by dance artists (Empowering Dance, 2020). The European Commission funded Empowering Dance 2018–2020 project identified soft skills that are used within contemporary dance practice with non-trained people. These skills included soft skills that are primarily introspective, such as patience, self-efficacy, self-perception, goal setting, critical thinking, as well as soft skills that are primarily inter-relational, such as conflict resolution, negotiation, active listening, empathy, dealing with uncertainty and complexity, taking care of others, flexibility and adaptability and understanding and appreciating differences (Empowering Dance, 2020). There is scant literature on soft skills and dance, with one review of the benefit of soft skills as part of dance pedagogy in China (Buck, 2022) and a research study on the use of soft skills in leadership development whilst practicing folk dance at a university in Peru (Bedoya et al., 2022). There has been one other European Commission funded industry-focused project on soft skills and dance (Moving into Soft Skills, 2024), led by a consortium of somatic practitioners. Its approach is to provide digital tools to help facilitators and educators explore the presence or absence of soft skills through using the body. Additionally, the British dance artist and coach Wookey (2024) led a small industry-focused research project (accessed 2024) that explored how the soft skills of dancers could be transferred to other professions. All of these studies point to the importance of the moving body when developing soft skills.

Additionally, notwithstanding excellent work on embodied agency in various fields and in soft skills there has been hardly any overlap between the concepts in Dance Studies, despite this being fertile ground. The closest the discussion comes is in the writing on embodied learning where there are examples of the importance of the body and physical practice in skill learning (see for example Zarrilli, 2004; Andersson and Österman, 2015; Anttila, 2016; Ravn, 2022). There is therefore a gap in knowledge and understanding of soft skills as it pertains to embodied agency through dance.

## The case study and approach

The impetus for my discussion is the dance industry-led project, of which I was part, mentioned above, Empowering Dance: The Soft Skills Teaching and Learning Approach (2020–2023), funded by the European Commission through Erasmus+ and involving as collaborators European Dance Houses, dance artists and community dancers from across the European Union.^1^ The goal was to help dance artists recognize and articulate their soft skills. This culminated in creating the online *Soft Skills in Dance: A Guidebook to Enhance your Practice* (Empowering Dance, 2022), marking the contribution of dance to the incubation and development of soft skills. The industry orientated guidebook, co-authored by myself and dramaturg Monica Gillette with the Empowering Dance consortium, was a series of tools and reflective tasks for professional dance artists to identify, articulate and develop their soft skills.

The collaborating team included five dance artists and four participatory dance groups. Unlike usual research projects, the dance artists and groups were not subjects of research but collaborators in developing the guidebook and contracted to do so.^2^ The research and development was characterized as participatory action research where dance artists led the community groups in weekly dance sessions and then had bi-monthly sessions of reflection, exchange, dialogue and analysis on soft skills development with myself, Gillette and the consortium evaluator who had also witnessed one third of the sessions. This happened over a period of 9 months, with the first 4 months of sessions conducted online due to COVID-19 pandemic lockdown situations in all the participating countries.

The sessions drew out themes from each dance practice, for example, themes of leadership, trust, vulnerability, taking care, and then related these themes to soft skills so the dance artists could identify and articulate them in their practice. Through this discussion, we discovered the enabling conditions necessary for the development of soft skills and the attitudes and values needed to engage socially, as well as focusing in on what dance practices specifically offer to make soft skills flourish and what approaches are needed so that they are recognized and communicated (Empowering Dance, 2022).

Throughout the project the dance artists' use of language and modes of communication with their groups and in their journals was analyzed. The process was dynamically reflective, where the individual dance artist was given space for experimentation and individual learning, which through dialogue, became the basis for generating shared knowledge.

One of the community groups, or case studies as they were called, was a longstanding group called Dance Well: Movement Research for Parkinson, or Dance Well for short. The core founding group is situated in the Civic Museum of Bassano del Grappa, Northern Italy, but for the case study it also used another of its sites in the Vento region, the Teatro Civico in Schio. These groups were already in existence and fluctuated in numbers depending on guests and month, but consisted of around 25 core members in Bassano and 15 in Schio. Of note, Dance Well's approach to dance and informal network has expanded in the last three years to different locations within Italy, Europe and Asia and is an influential approach in the field of dance for Parkinson's internationally.^3^ Dance Well promotes dancing in artistic contexts and within spaces of cultural heritage, such as museums. It is aimed primarily, but not exclusively, at people living with Parkinson's. In fact, the non-exclusivity gives it an intergenerational energy that envelops a larger section of the local community (including in Bassano del Grappa people seeking asylum housed there) than seen in other dance for Parkinson's groups. It is usual for several dance artists to contribute to facilitating the sessions, where movement improvisation and exploration is prioritized. A teacher training programme is also an important component.

In reflecting on what this specific case study brought up, I see a number of interesting elements that are pertinent to a discussion on embodied agency in relation to soft skills. The specific context of community dancers with Parkinson's helps me to elucidate a different understanding of embodied agency, one which is impactfully connected to soft skill development. The following discussion is not the results of empirical research into the connection of embodied agency with soft skills, but a reflection on this connection post-study in soft skills development. In laying out an argument that brings both concepts together, I provide an original approach to thinking about how it could be possible to discuss embodied agency in the context of socially engaged dance with people who have a neurodegenerative movement disorder. At the same time this essay provides a new examination of soft skills as they appear in dance practice and their potential for social inclusion and community building.

## Embodied agency in the professional dancer

The fluidity, adaptability and complexity of a dancer's movement are often used as examples of how dance at a level of mastery is embodied. The freedom this gives the dancer to choose what and how they move is enjoyed at a subconscious level, with often tacit knowledge driving the artistic and technical brilliance. There is a great accomplishment here that facilitates power and agency (Zarilli, (2000 [1998])). Dance scholar Levin (1983) studied the ballet dancer, a performer of virtuosic gracefulness. Levin argues that becoming seemingly weightless in the eyes of the spectator and playing with the suspension of time is the mastery of that art form. It is the “elite” dancer with many years and hours of experience that may accomplish these feats. It is only by inhabiting the technical form so completely without conscious thinking through that the mastery of the movement qualities can happen (Levin, 1983; Zarilli, (2000 [1998])^4^).

Actor trainer and theater scholar Zarrilli (2004) explains more. He uses the example of learning the Lion Pose in the Indian martial art Kalarippayattu to illustrate the embodied mastery of movement that occurs at a sub-conscious level. The first stage of learning is a conscious action, where one learns how to place one's feet, adjust the gaze and spine. Skills are acquired when actively, and reflectively, responding to the teacher's guidance as to how to adjust the placement of the body for effective movement. This skills acquisition in turn allows the performer to move to and from the Lion Pose to other movements seamlessly. The performer now knows how to use the Lion Pose and can effectively master the movement as it becomes intuitive:

The individual's proprioceptive sense allows one to make subtle, minor adjustments to the very act of placing the foot without thematizing the adjustment, i.e., one's bodymind “intuitively” adjusts as one moves (Zarrilli, 2004, p. 659).

Sociologist Shilling (2016) adds that mastery of a craft is also a matter of sensing differently, as well as cognitively and physically learning a system of movement. He argues that the senses are employed to guide the expert to problem solve when the unexpected happens; that the senses may guide the dancer, boxer or soldier to know how to adjust his or her body and its movement. The senses play an important part of the intuitive or “tacit” knowledge about navigating the world or stage when the expert is faced with new conditions or other movers. Philosopher Dewey (1925) termed tacit knowledge as “anoetic occurances”, namely “the ‘subconscious' of human thinking” (p. 299). He poetically explains:

we continually engage in an immense multitude of immediate organic selections, rejections, welcomings, expulsions, appropriations, withdrawals, shrinkings, expansions, elations and dejections, attacks, wardings off, of the most minute, vibratingly delicate nature. We are not aware of the qualities of many or most of these acts; we do not objectively distinguish and identify them. Yet they exist as feeling qualities, and have an enormous directive effect on our behavior.

He continues:

They give us our sense of rightness and wrongness, of what to select and emphasize and follow up, and what to drop, slur over and ignore, among the multitude of inchoate meanings that are presenting themselves. They give us premonitions of approach to acceptable meanings, and warnings of getting off the track (Dewey, ibid).

Or as Chris Shilling more succinctly put it, that knowledge, or more precisely that *knowing*, which exists “independently of conscious thought, embodied at a pre-conscious level as an awareness, intuition or practical ability” (Shilling, 2016, p. 1214). Dewey underlines the importance of anoetic knowing for intuitive judgements in action. In relation to professionals dancing, this has been characterized as “on-one's-feet, thinking-while-doing” (Bresnahan, 2014, p. 92) and manifested in, what dance philosopher Aili Bresnahan calls “improvisatory artistry” (Bresnahan, 2014, p. 85). The improvisatory artistry requires spontaneous, intuitive (yet grounded in experience) choice making that Bresnahan argues is agentic. In essence, the embodied agency afforded by experienced dancers allows them to move quickly, effectively and efficiently, as well as to create meaning and even wonder and awe for others through their actions.^5^ There are several terms in use currently for tacit knowledge. I shall use “anoetic knowing” as it is commonplace within several disciplinary circles, although it should be noted that some of the authors noted below prefer the term tacit.

I have already mentioned that experienced movers move with anoetic knowing. Anoetic knowing is particularly prevalent in embodied practices, such as dancing, where it is the knowing through doing which builds understanding and is learnt through practicing (Andersson and Österman, 2015). Please note the emphasis on actions/verbs in this definition. Not learnt through theory or conscious thinking alone, anoetic knowing is acquired through the process of regular action (Polanyi, 1967). In learning through experience and doing, the embodied understanding and knowing built up is nearly always captured and processed beyond the need for words and detailed description and categorization, as befits the acquisition of technical skills (hard skills). Andersson and Österman (2015) give a case study of a person learning to sail. In the example, although sailing requires a specific technical skill that can be learnt through a manual (a worded description), it is only by experimenting through movement and in the specific weather conditions that the sailor student discovers the gap in technical know-how and what is required to achieve a particular move or to surmount a specific challenge. Dancing in any technical form is the same. Watching any rehearsal, the observer will hear the teacher suggest tiny shifts of weight, positioning, gaze and feeling. The dancers have to experiment and sense what these “nudge words” of the teacher could form in their own body to create a journey toward the desired outcome. As Andersson and Östman note,

the idea that “the pupil must discover it for himself”…means that he or she has to learn to functionally coordinate his or her experiences to *create* intelligent action. When human beings respond to their internal and external environment they then have the possibility to expand their experiences and further their tacit knowing. In other words, they grow (Andersson and Österman, 2015, p. 277).

This process of anoetic knowing is gradually ingrained, not solely through following the manual but through experimenting with the body's own grasp of sense-making. Within a specific context where inflections of movement give sense and create meaning to those who inhabit that context, or institutional setting, the moving body is the site of learning (Dewey and Bentley, 1946; Polanyi, 1967; Gourlay, 2002).

Not only is anoetic knowing embodied within learnt action, it is also embedded within institutional—cultural—knowledge (Andersson and Österman, 2015). Anoetic knowing is not peculiar to an individual's internal processes but is often a shared understanding between people within an institution (or cultural practice). Despite the difficulty in articulating anoetic knowing, it is understood in the act of trying to do it, and those who practice a specific skill or action understand what that action is getting at. Anoetic knowing is a process that is meaningful alongside the embodied rules of action and cognitive beliefs adhered to by the people (for example contemporary Release-based dancers) who practice a particular skill (for example, contemporary Release technique) (Polanyi, 1967). This is important if anoetic knowing is to be characterized as sense giving and sense making (Polanyi, 1967).

Sense-making is not just about creating and developing meaning by giving form to the technical manual. It is about also bringing sensory and sensual contributions to the effort of sense-making through embodied action. Most scholarship on anoetic knowing is based around doing and practice. It is functional. However, it is important not to neglect the fact that embodied knowing is also affective because the senses are the vehicles from which the body is in communion with its internal and external worlds. The moving body creates sense through the sensory, sensuous and emotional resonance of its inflections. No more so than in the aesthetic realm of dancing where the emotional and sensorial power of the moving human is highlighted. Dance scholar Vida Midgelow points out that specifically the embodied knowing of a dancer is her sensuous and sensory movement experiences “be it her particular mode of physical knowing that comes from dance training or that which is found in felt, emotional, critical or memorial realms” (Midgelow, 2013, p. 3). The dancer draws on his or her own experiences in life to feed into the technical know-how of movement.

## Embodied agency in the community dancer

The topic of embodied agency in dance becomes more complicated when examining dance for the older person who has had little training, who may have joined a dance project organized for his or her community. Given the discussion above one could surmise that embodied agency may only become possible with many years of practice. Yet embodied agency is a key vision for socially engaged dance practices, particularly using improvisation. Perhaps to begin to explore this one might seize on Midgelow's description of embodied knowing above where sensory and affective experiences in life (and learnt culture) color the “doing” of movement.

Although there are two distinct areas of socially engaged arts practice^6^—one centered on anti-institutionalization and social destabilization, and the other on interdependent support and social imagining (Jackson, 2011)—I will concentrate on the latter where most socially engaged dance sits (Bannon, 2018). Socially engaged dance practices, including community dance, are those that involve communities and individuals at various decision-making levels, as collaborators, co-creators, or engaged movers. It is co-operative art making (Matarasso, 2019). Additionally, and because of the emphasis on sharing, socially engaged dance practice aims to be a “transformative and generative participatory practice, which enlarges what is possible in a territory or ecology. It supports processes of inclusion and cohesion, and is active in promoting aspects of social justice” (Empowering Dance, 2022). In other words, socially engaged dance broadens the aims of participatory or choreographic practices. It is concerned with building connection, relationship and community that ripples out from the dance practice into the places where it is hosted. As such, dance becomes a form of activism (Tate, 2024). Dance scholar Fiona Bannon writes that,

what citizen art, activist art, and participatory arts have in common is that they work to stimulate the active artistry and intellect of others. They tend to build infrastructure and connections that forge visible and sustainable bonds with people's worlds that endure beyond the political fashion of any given moment (Bannon, 2018, p. 100).

What Bannon alludes to in her last comment is that socially engaged art, and as she goes on to investigate, dance, will operate beyond the call for instrumentalization of the arts and their adherence to specific local or national governmental policy.

Embodied agency is important in the context of socially engaged dance because the body is at the center of the practice-as-activism. Dancing is the act of moving with and through the body. The body is also integral to a human's identity and mode of being, as well as being a site of traumatic, as well as joyful and mundane experiences and cultural inscription (Noland, 2009). To pick up on Midgelow's description, the lived experience is brought to bear in socially engaged dance practices, often more so than in artistic exploration by professional dance artists. Since the intention in these body-based practices is to build connection, community and relationship through shared decision-making, embodied agency becomes an intended consequence, at least theoretically.

I say theoretically, because although embodied agency becomes the important intention, there is still the challenge of realistically corporeally creating and inhabiting agency within a dance context.^7^ Embodied agency is not straight forward, but since it is a key intention in socially engaged dance practices, I would like to take a specific example to explore further what embodied agency could mean in that context, as well as what the conditions are that might make it work.

I would like to focus in on the amateur older dancer with the neurological condition Parkinson's. There are a rapidly growing number of dance programmes and initiatives for people living with Parkinson's around the world, some of which come under the umbrella of socially engaged dance practices and which I have studied over a number of years (see for key examples Houston, 2011, 2015, 2019, 2020a,b; Houston and McGill, 2013). As mentioned in the introduction, the dance group for people with Parkinson's I am focusing on in this discussion is Dance Well, Italy. It is a programme that exemplifies many of the values of socially engaged art and is not purely a series of dance classes taught to people with Parkinson's. It is strategically more radical than that. Dance Well provides dance sessions and events for people with Parkinson's and other citizens in the city, led by a rotating group of dance artists and situated in a site of cultural heritage. Using mainly improvisatory methods, the sessions increasingly encourage the dancers to make artistic decisions, gain confidence in their own way of moving and to connect with a diversity of people curious to dance with them. Bassano also holds artistic residencies for contemporary dance artists creating work and many of these artists are invited to also work with Dance Well, and even creating productions on the group. This is a longstanding group, who have been together for many years and yet continually welcome in itinerant artists, refugees and others to dance. It is of note that people with Parkinson's dance with a larger group of people without Parkinson's, which includes those who were in some other ways socially marginalized or minoritized. As this article will expand later, practicing soft skills through dancing did not just impact the embodied agency of people with Parkinson's, but others around them too. But whilst the discussion later will reference the wider group who are welcomed into Dance Well, I will emphasize those with Parkinson's in this section, as a way of giving focus to a specific example where embodied agency is often lost.

Parkinson's is a condition that gradually pushes away embodied agency: the “technique of the body”, to use Mauss's (1935) terminology, is often in daily life discordant and out of control. The mastery of every day movement breaks down. Parkinson's is clinically diagnosed through identifying two out of the three cardinal symptoms, bradykinesia, tremor and rigidity of muscles (Adams and Victor, 1993; McAuley, 2003). In addition, there are many other symptoms that can occur including postural instability (McAuley, 2003). Parkinson's increasingly affects the ability to walk normally and without falling over or freezing (getting stuck) (Allen et al., 2013). The condition can dimmish vehicles for communication, such as speech, writing and facial expression (Kim et al., 2009). There are many other symptoms that can occur in any one individual with the condition and these impact social participation and confidence in navigating the outside world (Solimeo, 2009; Houston, 2019). Particularly relevant to the topic of this article, Parkinson's gradually diminishes automatic movement—movement accomplished without conscious thought—as well as multitasking (Nieuwhof et al., 2017). The basal ganglia in the brain are the sites that create automatic movement and that are affected by Parkinson's. Journalist, Palfreman (2015), who has Parkinson's himself, likens the lessening of automatic movement to driving on a different side of the road to normal. Reflexes, operated from the basal ganglia, cannot be relied on and conscious thought from the cortex is needed to concentrate on the mechanics of driving. This slows a driver down and makes them more vulnerable to unexpected dangerous events on the road. The challenge of having to concentrate on movement that should be automatic makes its mastery even more difficult for people with Parkinson's.

One can conclude that the “doing” through old, anoetic knowing does not come easily, and less so as time passes. The experience of engaging with the world and with dance could be seen as “fractured” (Shilling, 2016) for the person with Parkinson's. Studies suggest that people with Parkinson's can still learn, but slowly and with substantial limitations (Nieuwboer et al., 2009; Marinelli et al., 2017), most notably in retaining new skills and learning that require attention and the use of cognitive strategy, rather than relying on pre-conscious thought (Marinelli et al., 2017).

Nieuwboer et al. (2009) note that cueing may aid in retaining learning, although the person may also be reliant on cueing to keep learning. Marinelli et al. (2017) note the positive effect of exercise on symptoms of Parkinson's, particularly those which do not respond well to medication or surgery, such as those affecting gait, posture and balance, and that furthermore exercise may further improve motor rehabilitation “by adding sensory stimulation, cueing, and music in pleasant social contexts and environment that increase task enjoyment”. Marinelli et al. quote Volpe et al. (2013), who have studied the effect of Irish folk dance on people with Parkinson's. To underscore his conviction, Volpe has notably collaborated with Dance Well for many years, inviting people with Parkinson's to dance at his clinic in the nearby Fresco Parkinson Center. Marinelli et al. (2017) observe that the strategies within the exercise activity will be important for accomplishment of the movement. These strategies include, as Nieuwboer et al. also point out, external cueing, feedback, as well as reward and motivation. They conclude that “this type of approach produce[s] good results probably by allowing the execution of correct movements under attention-volitional control, with a direct access to cortical resources and limiting the use of automaticity mechanisms that are affected by PD” (Nieuwboer et al., 2009). In other words, these strategies are seen particularly in dancing sessions where the facilitator demonstrates the movement and allows participants to copy them and where there is instant feedback and motivation. What this small body of research points to is that despite people with Parkinson's developing a skill learning deficit from quite early in the progression of the condition, exercise, such as dancing, might provide appropriate external help to enable people with Parkinson's to keep learning, or at least to accomplish new movement.

But despite including external cueing, feedback and reward, dancing does not normally get to the state of mastery that is described above. Additionally, it takes a very skilled facilitator to assign agency to a person that is being assiduously cued in movement. What is somewhat more useful to embodied agency is the fact that these tasks are enjoyable, bringing a felt sense of pleasurable purpose and motivation. I suggest, though, that to explore embodied agency further we need to look at this differently and in a new way. I propose that it is through nurturing soft skills within dance initiatives with people with Parkinson's that it might be possible to explain embodied agency in a socially engaged dance context with older people with a neurodegenerative condition.

## Soft skills and their development in the dance well case study

As noted at the beginning of this article, soft skills are patterns of thought, behavior and communication that help people navigate their own emotions and interpersonal relationships. Yet in the research leading up to the creation of the guidebook we found that dance artists were often not aware of their soft skills nor how to articulate them, even though they were using them in their dance practice (Empowering Dance, 2022). In revisiting Dewey (1925, p. 299) characterization of anoetic, pre-conscious knowing—“they give us our sense of rightness and wrongness, of what to select and emphasize and follow up, and what to drop, slur over and ignore”—it flags the intuitive judgement calls and behaviors that allow us to be openly present in a space with others, which also characterize the practice of using soft skills. Perhaps it is no surprise that dance artists use anoetic knowing in their practices with others, and yet cannot identify or articulate that knowing. Practicing soft skills is also to involve anoetic knowing.

So it is not surprising that soft skills have not been articulated much within dance scholarship or practice. Operating mainly on the level of embodied, anoetic knowing, soft skills have been difficult to translate into words or through analysis. It is perhaps also confusing that, as we discovered in the Empowering Dance project, soft skills overlap to form clusters, where one soft skill needs several more to be executed. Moreover, soft skills exist as the sub-layer beneath more specific technical skills that are more overtly practiced, investigated and spoken about. Despite the technical and artistic skills also being subject to anoetic knowing in dance (see above), the interest in trying to articulate these is greater; undoubtedly because they are specific to dancing and hold long traditions of debate, artistry and scholarship. Soft skills are general. They are not specific to any field and therefore do not hold the same interest to discipline-specific enquiry. Yet dancing does illuminate an important aspect of soft skills, namely that many of these soft skills come to life through embodied, sensorial action. The Empowering Dance project (2020–2023) concluded that:

“Dancing with others is an important pathway for developing soft skills, because the body plays a central role in how we navigate our emotions, responses and actions: soft skills grow through, with and embed in the body and by encountering other bodies in space” (Empowering Dance, 2022). The body is not often named in manuals of soft skills development, and yet it holds much importance for soft skill development.

I will discuss the practice of soft skills in dance to achieve a sense of embodied agency through the case study of Dance Well, as I have already mentioned. I have engaged with Dance Well in my writing before (Houston, 2019), but not in an academic paper to talk about its relation with soft skills. As I indicted earlier, the Empowering Dance project took on as a key element in the R&D process an internal reflexive dialogue with dance artist members of the group reflecting with guidance and prompts on their experiences leading, which led to discussion on the identification and articulation of soft skills. The dance artists leading Dance Well were Giovanna Garzotto and Elena Sgarbossa and a few elements stand out from their facilitation of the sessions. First the sense of warm invitation, of welcome, that every person had. This was coupled with awareness by the dance artists of the dancers' needs that day. The sense of inclusion was high. Second the use of imagery and the imagination in promoting certain qualities, dynamics of movement and motivations to move. Third the invitation to be curious about movement, time and space was taken up enthusiastically by the dancers. I will explore each element in turn in relation to how they developed soft skills in both the lead dance artists and the participating dancers.

In the Empowering Dance project, we discovered that there were some attitudes displayed by the dance artists that were modeled by the participating dancers and that led to the cultivation of soft skills within the group. We called these attitudes “enabling conditions” (Empowering Dance, 2022). In Dance Well the two most striking enabling conditions were making everyone feel welcome and creating a space where no one was judged for how little or how they moved. The welcome was enacted through words—greeting each person as they came in and finding out how they were—but also through actions, such as a hug, a clasping of hands, a touch on the back or shoulder, blowing kisses, and through smiling. The welcome went further in that it was reiterated at moments throughout the sessions. This happened by instructions to meet the gaze of someone across the room, by greeting a person walking past in the space, or by holding hands, and by the invitation to stay afterwards in conversation. Since the first four months of the sessions occurred during lockdown periods in the COVID-19 pandemic, these were conducted online. Despite not being able to touch, the instructions were similar. The group were asked to bring their gaze, their smile, or their moving hands, close to the screen, to be in contact with their fellow dancers online.

Comments during dancing, such as “I'm tired, but I am smiling”, acknowledged the reality of the fatiguing body of the Parkinson's dancer at the same time as offering an alternative happier vision through dancing; an inner smiling body. In one online session with the Schio group Garzotto invited people to find a sofa or comfortable floor space. The group were invited to create an imprint of their body where they lay and to explore what happens to the changes in way the body is organized when one puts a foot up, or an arm. In subverting the hierarchy of being upright, which is not necessarily a comfortable position always for a person with Parkinson's, the reclining aware body is celebrated as capable and reflective.

The dance artists noticed that not only did the participant dancers respond to the warmth of the welcome, they also started modeling the same welcoming behavior themselves toward others. This included in Bassano welcoming a group of young asylum seekers from North Africa to dance. Housed in a city with a far-right local government, the asylum seekers were not welcomed universally. Dance Well participant dancers did model the welcome they had received from the teachers to the young people, in voice, action and attitude. A couple of the young people eventually became integrated members of the teaching team.

Creating a welcoming, non-judgemental space enabled the soft skill of understanding and appreciating differences to be brought into focus. Also named as “respect for diversity” by Hoskins and Liu (2019), this soft skill is seen as important for active citizenship. Diversity is defined in several ways. For example, the Council for Europe's 2016 report talks of cultural diversity in its list of competencies (also known as soft skills) for living in a democratic society. It writes: “This value is based on the general belief that other cultural affiliations, cultural variability and diversity, and pluralism of perspectives, views and practices ought to be positively regarded, appreciated and cherished” (Council of Europe, 2016, p. 12). In the context of Dance Well, it is important to add in diversity as it pertains to health, bodily function and mobility. I would argue that it is also the case that this soft skill was accompanied and demonstrated by the soft skill of taking care of others.

Dance Well welcomed people with Parkinson's and those without, or with other health challenges. Individuals therefore had to negotiate encounters with others of varying movement capability and health. Additionally in welcoming young asylum seekers the group had to move with those from a different culture and political status, as well as from a different generation. Inevitably, health, cultural, ethnic and political differences are inscribed in the body, how it is perceived by the self and others, and how it is dealt with by institutions (Noland, 2009). The social marginalization of those with neurodegenerative conditions, of those without citizenship, and those of a minoritized culture or ethnic background is not to be underestimated. Embracing of bodily difference is uncommon, yet much needed for those who have to deal with judgement, prejudice and lack of institutional support.

The fact that much difference is related to the body means that dancing in relation to others and with care potentially holds a key to connecting and even fostering a sense of belonging in the face of diversity. To do this requires dancing to become a relational, people-centered activity. By this I mean that the act of moving with others in the space intentionally prioritizes connection and the purpose of the act is primarily focused on the people in the room, rather than on the technicality of the form of movement. The soft skill of understanding and appreciating difference was practiced with care in Dance Well bringing with it the outcomes of connection and a feeling of belonging.

The key outcomes of connection and belonging, were indicated in several ways and encompassed several soft skills, as well as understanding and appreciating difference. First through creative and collaborative movement tasks, for example in creating a score together to improvise around. Such tasks demand soft skills of cooperation, team work and negotiation and so also an understanding of perspective of those they negotiate and cooperate with. Second through communal decision-making, such as inviting the Dance Well dancers to commission a choreographer from Bassano's Operaestate Festival to make work on them. Such decision-making is a key artistic contribution to the festival and indicates not only critical awareness of aesthetic preferences (again a soft skill), but also membership of the artistic curation team. Third through actions and gestures that state each person is welcome here, as outlined above; and lastly through cultivating a sense of pleasure and joy in moving together. It is no co-incidence that the first research to come out of another Parkinson's dance group, Dance for PD, concluded that one of the most important aspects of dancing with Parkinson's was embracing joy (Westheimer, 2008). Similarly to Dance for PD, pleasure is evident in Dance Well through movement tasks that allow people to play, to have fun within a movement conversation, and to explore the sensuous nature of the living, sensate body outside of an everyday context where their bodies are seen as flawed. This invariably calls upon the imagination, a key ingredient of playful movement. For example, one session started up a movement conversation through the blowing of kisses to one another. This was followed, as the catalyst for movement improvisation, by the dancers imagining clouds transforming in response to the movement of their bodies. The playful, imaginative component of the classes allows the dancers to not only create their own unique ideas that give them the capacity to move with expression, but also to share those ideas with others who are responding in their own way to the same prompt. This then creates moments of movement dialogue where each imaginative idea is connected to another through embodied movement.

The creation of these pleasurable connections, as well as collaborative aesthetic decision-making, are the components that develop an environment where differences are appreciated and belonging to the group is fostered. Social justice activist and philosopher Ginwright (2022), p. 15 argues that “belonging is the capacity to see the humanity in those that are not like us and to recognize that the same elements that exist within them also exist in us”. On a level of playing through and with movement, I argue that it is possible to see humanity in those we move and play with, to make sense of and with them. For example, by taking the movement of the dancers with Parkinson's out of a context where it is pathologized and where the dancers are separated and “othered”, and into a situation where the imagination inflects movement and expression, the whole person is more readily seen and recognized; particularly because each person is sharing their own response to the imaginative impulse through embodied movement. It is important to note that the sharing and recognition is reciprocal. Each dancer with Parkinson's is not merely being seen but are recognizing and appreciating others in turn. Meeting others in improvisational, imaginative movement may spark new sense-making not just for that one actor, but for all participating (Merritt, 2013). In bringing their own responses through movement, the dancers may together create new ideas and sense and become collaborating agents.

What I am also describing above could be the conditions for the development of the soft skill of empathy through dance. As Hoskins and Liu (2019) point out in their UNICEF report on life skills, empathy is a contested term spanning several disciplines. Bringing common elements together, Hoskins and Liu summarize that definitions of empathy contain both “a cognitive component of understanding the feelings of another by imagining his/her perspective and situation” and an affective dimension “in which individuals can reproduce these feeling in themselves and simultaneously realize that these feeling are not their own”. They go on to explain that “as a result of these internal processes, individuals are able to sympathize and to act to support the other person” and, they add, with emotional control (Hoskins and Liu, 2019, p. 64). Dance Studies emphasizes the embodied and kinesthetic dimension to empathy, with Foster (2011), Warburton (2011), Reynolds and Reason (2012) as some of the key contributors, with accompanying neuro-scientific papers such as Calvo-Merino et al. (2008).

The generosity of the Dance Well dancers toward each other, indicates potential empathic support being given by individuals. Yet in examining the sessions the soft skill of empathy is also created within the dancing itself. Dance artist Giovanna Garzotto articulated her experience and the specific inflection of empathy within Dance Well:

The point of empathy in a Dance Well class is not understanding but being available to experience with someone else. As a Dance Well teacher [I] experience a sense of going beyond my own body. Empathy is when the borders of my body expand and go beyond. It is a very physical experience to me. It can be recognizing that we share the same speed intensity based on a proposal or that we have reached the same level of engagement (Garzotto, in Empowering Dance, 2022).

Garzotto continues:

It's difficult to conceptualize this experience. But there is a task that can be a good example. It's the mirroring task. It is the first improvisation task that we propose to newcomers and new groups of dancers because it triggers movement, and it breaks loneliness. But we then realize it also activates active listening, empowerment, co-leadership, and breaks the barrier between bodies even without touch. If I think back to the first years of Dance Well, we used this practice because it was the easiest one available. We then experienced through practice how many soft skills it activates, including empathy (Garzotto, in Empowering Dance, 2022).

Garzotto's experience fits appropriately into the philosophical discussion around dance and empathy. Taking cue from Bourriaud's (1998, p. 24) argument that “art is a state of encounter” and relational, the act of dancing can be seen as movement between bodies (Reynolds, 2012), which produces affective sensations in dancers and spectators alike (Thompson, 2009). Taking this as the case, the discussion around empathy in dance is primarily in relation to affect (to affect and be affected). Reynolds (2012) characterizes affect as “fluid relationality” (p. 127). It is less about connecting between discrete beings, as posed in Hoskins and Liu's summary, and more about sharing a liveness and dynamism across personal boundaries. Theater scholar Thompson (2009, p. 119) suggests that affect is a “capacity and intensity”. It is an aliveness and vitality within engagement with others and importantly affect refers to “the augmentation or diminuition of a body's capacity to act, engage and to connect” (Cough and Halley, 2007, p. 2). The body's capacity to act is seen in Garzotto's examples where she describes her body going beyond its boundaries, to expand, and to attune to the experience of another, which moves her and her partner.

The mirroring task, which Garzotto describes, asks one person to follow the other's improvised movements. Each take turns to being the leader. It is a task that is also used in Dance Movement Therapy and has some investigation around its significance in inducing empathy (see for example, McGarry and Russo, 2011; Mintarsih and Azizah, 2020). Yet here it is used within an artistic context where it is the participation in an artistic generative practice that is foregrounded. Receiving and generating movement that requires active listening to “tune in” to one's partner also causes, in Garzotto's evocative words, expansion. Whilst usually related to professional dancers and to an inward focus (Batson and with Wilson, 2014) attention in this case refers to the sensory attunement to another's capacity to move. The expansion experienced through active listening (another soft skill) can change perception and perspective of both movers. Philosopher Brian Massumi explains:

When you affect something, you are at the same time opening yourself up to being affected in turn, and in a slightly different way than you might have been the moment before. You have made a transition, however slight. You have stepped over a threshold. Affect is this passing of a threshold, seen from the point of view of the change in capacity….A body is defined by what capacities it carries from step to step. What these are exactly is changing constantly. A body's ability to affect or be affected—its charge of affect—isn't something fixed (Massumi, 2015, p. 4).

The soft skill of empathy, developed in these generative, relational and cooperative movement acts points to a collective practice of gentle action and agency. For a person with Parkinson's the changing of one's capacity through affective empathy might be important, even empowering. Despite the inevitable threat of more limited movement, a Parkinson's dancer can change the capacity to affect and be affected through dancing, not because they have Parkinson's—there is no pity involved—but because they choose to stand within a relational movement practice with others.

Now that I have pointed out how one might conceive of soft skills within a socially engaged dance practice with and for people with Parkinson's, I would like to skirt back to the discussion initiated at the beginning of this article on embodied agency and mastery of movement. What linked that part of the discussion with the outlining of soft skills in a relational dance practice is anoetic knowing. What the study of soft skills generated through a physical practice suggests is that just as the mastery of movement is embodied, so too are these relational skills, crucial to a person's engagement with others and his or her environment. In contrast to the embodied agency afforded by the mastery of movement, soft skills might be what is practiced and honed by those with little formal dance training, including those with a neurodegenerative movement disorder.

I have spoken above about the embodiment of soft skills, and their potential to change perspective. The potential to change perspective of the actor and those he or she comes into contact is, I would argue, agentic. I described Dance Well as a programme that is radical in its strategy to link various artistic and cultural institutions to the dance classes, as well as the people who live, visit and seek shelter in the city. Within the dance sessions themselves there is also an enthusiasm to give those that dance more decision-making within what and how they dance. I term this radical because for a dance programme for people with Parkinson's it unusually sets itself up not just as a tool for wellness, but as a community building and artistic development hub of dynamic movement research by those who dance there. “Movement research” is a term that Dance Well uses itself to describe the improvisational, generative exploration that happens through movement within the sessions. Generative exploration is not about standing still. Sociologist Chris Shilling's view of embodied agency is pertinent to Dance Well's social position. He argues that it is important to

require a view of the embodied dimensions of agency that is shaped by the social system but is no mere reflection of it; that possesses a creativity able to affect the reproduction or transformation of social structures; and that is subject to change over time (Shilling, 1997, p. 748).

Similarly, Noland's (2009) definition of embodied agency cited at the beginning of this discussion stresses the power to change behavior or beliefs to react or collaborate, to resist or innovate. It is with this characterization of embodied agency in mind when I think of Dance Well's ability to create a radical proposal for movement within specific social and artistic structures and systems that do not normally engage with people with Parkinson's, or for those without citizenship. The soft skills present in Dance Well's work create the groundwork for a step away from systemic marginalization and disempowerment.

This article makes the claim in relation to Dance Well that physical, aesthetic practice with others may build sensory, affective experiences, which in turn develop qualities of care, appreciating difference, empathy, active listening, among others. These experiences are sense-making on the level of building and sharing a capacity to act and step “over a threshold” (Massumi, 2015, p. 4). It is through this shared anoetic sense-making that Dance Well dancers may transform not only their own positioning regarding their sense of capacity with Parkinson's, but also that of others who dance with them. Practicing soft skills through dancing might be an important step to embodied agency where change by dancing, through dancing and in dance happens. In Shawn Ginwright's ground-breaking book *The Four Pivots* on reimagining social justice, he argues that transformative relationships focus on what I would term soft skills and those qualities seen in dance improvisation that build up the practice of soft skills. He writes that transformative relationships

are based in those features of life like care, vulnerability, love, curiosity, connection. Transformative relationships are formed when we exchange pieces of our humanity with each other. When we do that, we give permission to others to do the same (Ginwright, 2022, p. 115).

Dance anthropologist Grau (2013) argues that it is dance's special relationship with embodiment that makes it an effective medium for the recognition of each other's humanity and “a favorable medium for dealing with issues of social conscience”, as seen in Ginwright's work in social justice. What Ginwright describes can be seen in the empathetic expansion of the body within the mirroring task, the embracing of the older woman who can no longer walk without assistance, the young woman from Ethiopia, the middle-aged man who is unsure of how he will cope with his new diagnosis of Parkinson's, the embodied sharing of imaginative proposals. The embodiment of soft skills within movement improvisations opens out the landscape of possibility for every one of the dancers and those around them and in doing so brings agency.

## Future directions

To summarize, the article has drawn together two distinct areas of enquiry, that of embodied agency and soft skills within the field of community dance. My case study of people with Parkinson's dancing demonstrated how soft skills could encourage embodied agency whilst dancing. Whilst Dance Well was a concrete example, there is no prescriptive method for working with soft skills in dance for future studies and practice. Instead, there is a value system that will prioritize person-centered facilitation, a goal to set up the enabling conditions seen above to help fertilize soft skill development, and a commitment to creating movement investigation that enables participants to choose and imagine their own movement worlds. There is deliberately no prescription to developing soft skills in dance because to do so would close down different avenues of movement possibility and engagement suitable for diverse groups and different artistic practices developed by dance artists and those they work with.

In thinking about future directions for researching soft skills and embodied agency in dance, it would be interesting to consider more tangibly the notion of social justice and transformation suggested, which would need a long-term study. Another future direction would be to examine in more depth the connection between anoetic knowing and soft skill practice, as well as the mechanism between agency and imagination. Whilst this paper has established the link between embodied agency and soft skills practice in dance, there is plenty more empirical and philosophical study that can be mined in this field.

This article has argued that practicing soft skills has huge potential in a dance context to create an environment where embodied agency is present for participants with Parkinson's, as well as for others who dance with them. Although my example was primarily focused on people with Parkinson's, the mixed group context suggests that working through soft skills in dance may positively affect any population or group, for example, teenagers, bankers, health professionals, social workers. Indeed, the Empowering Dance project went on to work through movement with social workers and teachers in Italy. Crucially, what my argument has shown is that those who have a health condition, illness or disability, who might be denied respect and dignity in everyday life, might, through the practice of soft skills dancing, develop embodied agency. It points to the importance of creating training for dance artists in recognizing soft skills they have or want to develop in their own practice. It highlights the expansive, generous nature of an art form that can accommodate soft skills practices and development for not just professionals working in the field but for the transformative potential they have for everyone on the dance floor.

## Data availability statement

The original contributions presented in the study are included in the article/supplementary material, further inquiries can be directed to the corresponding author.

## Ethics statement

Ethical approval was not required for the studies involving humans because permission was given and an ethical process detailed through legal contracts to R&D collaborators drawn up by industry partners. A robust on-going ethical process and data management was managed by the lead industry partner. The case studies were conducted in accordance with the local legislation and institutional requirements. The participants provided their written informed consent to participate in this study.

## Author contributions

SH: Writing – original draft, Writing – review & editing.

## Footnotes

^1^Funded by the European Commission, Erasmus+ programme, the Empowering Dance consortium consisted of Kampnagel/K3 Tanzplan Hamburg (lead), La Briqueterie CDCN Val de Marne, CSC Bassano del Grappa, Dansateliers Rotterdam, University of Roehampton London, Croatian Institute for Movement and Dance Zagreb, University of Zagreb.

^2^Ethical consideration and permission were managed through the written consent, care and data protection processes and policies, and legal contracts drawn up by each consortium partner who organised the dance sessions and hired the dance artists for the project, as well as being overseen by the project lead partner Kampnagel for the consortium as a whole. Ethical action was an ongoing process of checking in with collaborators, of “held” conversations with Kampnagel's dramaturg and evaluating the systems and processes at 3 monthly intervals.

^3^Dance Well International's network reaches Italy, France, Germany, Lithuania, Czech Republic, Hong Kong and Japan. Another, much larger international network, with certified teachers and groups, is Dance for PD. Dance for PD is based in New York and has many hundreds of affiliated members around the globe and operates a slightly different model of working to Dance Well.

^4^This view is seen as current thinking in dance, but seen especially, for example, in discussion with dance scholars using Bourdieu's theory of habitus (see for example Crow, 2020), or theories of improvisation (see for example Albright and Gere, 2003), or even as in McKinney et al. (2009) with AI and dance.

^5^This is not to say that expert movers do not think in action. See Montero (2016) for a cogent explanation, and also discussion of Dewey's work on the connections between noetic and anoetic knowledge in Shilling, 2016.

^6^Socially engaged arts practices are held within the umbrella term participatory art, which has more variety of definitions and political stances, as laid out by Bishop (2012). Although definitions and political traditions cross art form, dance has its own trajectory, ideologies and journey.

^7^The challenges of reaching the aims or ideals of community dance have been pointed out by Houston (2005) and Wise et al. (2020).
